# Boredom and affective temperaments as factors hindering smoking cessation: An exploration within an Italian sample

**DOI:** 10.3934/publichealth.2025003

**Published:** 2025-01-03

**Authors:** Fiammetta Iannuzzo, Michele La Versa, Fabrizio Turiaco, Gianluca Pandolfo, Carmela Mento, Maria Rosaria Anna Muscatello, Antonio Bruno, Clara Lombardo

**Affiliations:** 1 Department of Biomedical and Dental Sciences and Morphofunctional Imaging, University of Messina, Messina, Italy; 2 Department “Scienze della Salute”, University of Catanzaro, Catanzaro, Italy

**Keywords:** smoking, smoking cessation, affective temperaments, boredom

## Abstract

**Background:**

Smoking cessation presents challenges influenced by neurological adaptations and psychological factors, potentially exacerbated by susceptibility to boredom and affective temperaments.

**Methods:**

This study enrolled 409 participants via an online survey distributed among the Italian population through mailing lists, social networks, and messaging apps. Specific questions assessed cigarette smoking, while the Temperament Evaluation of Memphis, Pisa, Paris, and San Diego (TEMPS-A) and Boredom Proneness Scale (BPS) explored affective temperaments and susceptibility to boredom, respectively.

**Results:**

Results indicated smokers exhibited higher cyclothymic temperament scores compared to no-smokers and ex-smokers, suggesting a connection between this temperament and smoking behavior. Furthermore, the analysis demonstrated variable influences of specific temperaments on boredom proneness.

**Conclusions:**

These findings emphasize the significance of incorporating affective temperaments and boredom proneness into smoking cessation interventions. Understanding the interplay between affective temperaments and boredom proneness can guide the development of innovative and personalized cessation strategies. Further research is warranted to delve deeper into these relationships and their implications for intervention approaches.

## 1. Introduction

Tobacco is the second most widely consumed psychoactive substance worldwide, with over one billion individuals engaging in its use [1] and remains the leading cause of premature death before the age of 70 in Europe and in the United States, although its decline in the higher sociodemographic countries [1]–[4]. According to data, smoking prevalence is higher in lower education and income population groups [5] and among people with mental health problems [6], showing three times higher rates than in the general population [7].

In high tobacco consumption countries, smoking individuals who do not have tobacco-related diseases seem less inclined to quit [8]. The challenge of achieving smoking cessation may be attributed to both neurological adaptations derived from prolonged nicotine exposure and psychological mechanisms formed during previous attempts to quit smoking.

As for other abusive substance, abrupt discontinuation of tobacco induces a withdrawal syndrome characterized by irritability and anxiety, low mood or boredom, concentration difficulties, altered hypnic profile, and increased appetite, causing smoking cessation to be extremely challenging [9]–[11].

Several studies suggest that individuals may persist in smoking because they perceive tobacco consumption and nicotine exposure as coping mechanisms to alleviate feelings of emptiness and apathy linked to boredom [12],[13], or, in case of adolescence, to experiment or to emulate peers [14],[15]. The perception of boredom, described as the perception of environmental invariability or under-stimulation [16], encompasses two concepts: Situation-dependent boredom, termed “state boredom” (transitory), and situation-independent boredom, termed “trait boredom” (dispositional). The concept of “state boredom” highlights the influence of the environmental factors in creating conditions for a reactive boredom, triggered by external situations perceived as uninspiring, uninteresting, or repetitive; with the supposition that the cessation of the external trigger leads to the conclusion of the state of boredom. The concept of “trait boredom” seems to focus on the individual, whose innate trend results in a predisposition to boredom, creating a persistent susceptibility to monotonous environmental conditions [17]. There is some evidence that boredom in general is involved in mechanisms of cognitive (e.g., cognitive rigidity, distraibility), emotional-motivational (e.g., negative affect, decreased persistence, lack of motivation and apathy), and impulse-control maladaptation or dysfunction [16],[18]. Boredom can be considered a trigger for many forms of addiction, since a person is bored and may be tempted to seek out activities that offer immediate enjoyment or pleasure. There is evidence suggesting a concomitant increase in addiction and the experience of boredom among the population [19]. Based on some literature, it can be inferred that susceptibility to boredom might correlate with the challenge of quitting smoking, as it could serve as a notable predictor of smoking cessation outcomes [20].

Moreover, individual behavioral characteristics may contribute to the effective attempt to quit smoking; for instance, many researchers have investigated the relationship between affective temperaments and different types of addiction (including nicotine addiction) and the connection between specific temperamental traits and the inclination to use tobacco has become clearer [21],[22], along with personality factors as influenced element in the process of smoking initiation and maintenance [23].

In light of this, it could be appropriate considering temperamental differences in the topic of smoking cessation in order to increase effective and targeted therapies.

Temperament is the innate predisposition to respond to the external stimuli in a defined way; this construct relates to Kraepelin (1921), that described the maniac (hyperthymic), irritable, cyclothymic, and depressive temperament.

Over the years, the increasing attention to the role of affective temperaments on mental illness had led to the definition of different measurement tools. Akiksal and colleagues, with collaborators in the Menphis-Pisa-Paris-San Diego research group, proposed a semi-structured interview for affective temperaments, called TEMPS-A [24].

TEMPS-A was developed to measure affective temperaments based on the concept of core personality traits and it furthers our understanding of temperament types and their connection to mood disorders as well as other mental and physical health conditions. Therefore, it contributes to a more comprehensive psychological profile of patients, and it might support in prognostic assessments of smoking cessation, as indicated by López-Torrecillas and colleagues [25], who found that TEMPS-A can evaluate specific temperament dimensions linked to smoking behavior and outcomes in cessation efforts, while personality, broadly, seems to be a powerful predictor of current smoking, smoking initiation, smoking progression, and smoking cessation [26].

The following affective temperaments are described by a TEMPS-A questionnaire:

- Depressive temperament: When an individual tends to be pessimistic, self-critical, with a low self-esteem and prone to feelings of sadness and hopelessness, lacking energy, and enthusiasm.

- Cyclothymic temperament: When an individual experiences frequent and intense mood swings that are less severe than those seen in bipolar disorder, with a rapidly shifting mood from elation to depression.

- Hyperthymic temperament: When an individual has a consistently elevated mood and high levels of energy, with the tendency to be optimistic, sociable, and extroverted.

- Irritable temperament: When an individual feels frequent and intense episodes of anger and frustration, within the tendency to be annoyed and quickly reactive with hostility.

- Anxious temperament: When an individual is prone to excessive worry and fear, anticipating negative outcomes.

Understanding the temperaments can be informative in defining strategies for users to achieve smoking cessation: Each temperament trait may need to be addressed differently during cessation.

Considering boredom as a factor that may hinder smoking cessation, our purpose of this study is to frame the role of affective temperaments as predictor of boredom proneness in smoking cessation.

## 2. Materials and Methods

Data was collected between April 2023 and October 2023 via an online survey using Google Forms. The link was disseminated through mailing lists, social networks, and messaging apps to Italian population. The research method prevented incomplete responses by not allowing progress if any questions were left unanswered. To be eligible for the study, participants must not have any psychiatric disorders, significant concurrent medical illnesses, a history of alcohol or substance addiction, mental retardation, or organic brain disorder. We determined the exclusion criterion by asking specific questions to assess the presence of these conditions; for example: “Have you ever been diagnosed with a mental illness?”, “Have you ever been diagnosed with any form of systemic diseases such as diabetes, heart diseases?”, “Have you ever been diagnosed with alcohol use disorder?”, “Have you ever been diagnosed with a substance use disorder?”, “Have you ever been diagnosed with an organic brain disorder, such as dementia or traumatic brain injury?”.

The study was conducted with healthy subjects in accordance with the Helsinki Declaration. Participants were assured that their answers would remain anonymous and were collected in aggregate form to ensure the accuracy of their responses; informed consent was obtained at the time of the original data collection. Ethical approval was not required.

### 2.1. Measures

Cigarette smoking was assessed with the following questions: (1) “Are you a smoker?”. (2) “How many cigarettes per day in general?”. Participants were asked about their consumption of tobacco and classified as currently smokers, no smokers, and ex-smokers; defined in the following way; smokers were individuals who currently smoke tobacco products such as cigarettes, cigars, pipes, or other forms of smoking tobacco, within a specified recent period (the last month), ex-smokers were individuals who have smoked tobacco products in the past but have since quit from at least 1 month; non-smokers were individuals who have never smoked tobacco products.

Also, the survey assessed the age of smoking onset and the mean of attempts to stop smoking.

To achieve our goals, the following psychological tests were administered:

- Temperament Evaluation of Memphis, Pisa, Paris, and San Diego-Auto questionnaire version (TEMPS-A). The Italian short version of the assessment was deemed more appropriate for large-scale use compared to the original version, which consisted of 110 items. It consists of 39 items with dichotomous Yes/No responses, enabling the evaluation of affective temperaments across five subscales: cyclothymic (items 1–12), depressive (items 13–20), irritable (items 21–28), hyperthymic (items 29–36), and anxious (items 37–39). These temperaments are seen as subclinical traits that may predispose individuals to specific types of mood disorders. Reliability as measured by Cronbach's α was good for all TEMPS-A subscales (>0.70) [24],[27].

- Boredom Proneness Scale (BPS) is a psychometric instrument developed to measure the tendency to boredom, consisting of 28 items ranging from 1 (strongly disagree) to 7 (strongly agree). The scale items are designed to measure various aspects of boredom tendency, including attitude towards time, mental activity, emotional activity, and perception of repetitive or monotonous activities. The scale consists of three factors: ‘Internal stimulation-challenge’, ‘Apathy’, and ‘External stimulation-creativity’. A high score in all items of the scale is indicative of a high propensity for boredom. The alpha coefficients can be considered acceptable for all three scales: *α* = 0.70, *α* = 0.71, and *α* = 0.63. The Cronbach's alpha for this scale is 0.75 [17].

### 2.2. Statistical analysis

The statistical analysis was realized with the Statistical Package for the Social Sciences (SPSS) 25.0 software (SPSS Inc., Chicago, IL, USA). Descriptive statistics were used to summarize the data obtained from the study. Continuous data are expressed as the *mean* ± *SD* (standard deviation) and significant differences among groups were assessed using the one-way analysis of variance (*ANOVA*) with post hoc comparisons (Bonferroni). A linear regression analysis, in which the BPS factors (Internal Stimulation Creativity, Apathy, External Stimulation Challenge) were considered dependent variables, and age and all the TEMPS-A subscales were included in the equation (as independent variables), was performed to assess what kind of temperamental dimension could play the role of specific predictor towards the boredom proneness. The results were considered significant at a 5 % level.

## 3. Results

A total of 496 complete surveys were received: 87 were excluded according to the selected exclusion criteria. A final sample of 409 participants were identified in three subgroups: Smokers (*n* = 177; *M*: 67; *F*: 110), Ex-smokers (*n* = 63; *M*: 16; *F*: 47), and Non-smokers (*n* = 169; *M*: 52; *F*: 117). The clinical-demographic characteristics of the study participants are shown in Table 1: age among the variables examined was statistically significant among the three subgroups of participants, i.e., among smokers and ex-smokers.

Table 2 shows statistical analyses of the psychometric instruments applied in study participants. Statistical analyses (ANOVA and Bonferroni's post-hoc test) showed that smokers, compared to non-smokers, had worse scores in cyclothymic temperament [*F*(2) = 12.56, *p* < 0.0001; Bonferroni's post-hoc test: *p* < 0.0001], and, in addition, smokers compared to ex-smokers had higher scores in cyclothymic temperament [*F*(2) = 5.60, *p* < 0.0001; Bonferroni's post-hoc test: *p* = 0.025].

BPS factors (BPS “Internal Stimulation Creativity”, “Apathy”, and “External Stimulation-Challenge”–as dependent variables) and age and all TEMPS-A subscales (Cyclothymic, Depressive, Irritable, Hyperthymic Anxious, as independent variables) were analyzed in three linear regression models, as reported in Table 3.

Results from the regression analysis indicate that the predictor models account for 23.4%, 37%, and 23% of the total variance respectively in BPS “Internal Stimulation Creativity” (*F* = 14.3; *df* = 5; *p* < 0.0001), “Apathy” (*F* = 27.8; *df* = 5; *p* < 0.0001) and “External Stimulation-Challenge” (*F* = 14.0; *df* = 5; *p* < 0.0001) factors. The regression analysis evidenced that Depressive temperament was a negative predictor of the “Internal Stimulation Creativity” (*β* = −0.232; *p* ≤ 0.0001) and conversely was a direct predictor “Apathy” (*β* = 0.292; *p* ≤ 0.0001). Cyclothymic temperament was a direct predictor of “Apathy” (*β* = 0.271; *p* ≤ 0.0001) and “External Stimulation Challenge” (*β* = 0.365; *p* ≤ 0.0001), and Hyperthymic temperament was a direct predictor of “Internal Stimulation Creativity” (*β* = 0.376; *p* ≤ 0.0001) and “External Stimulation Challenge” (*β* = 0.176; *p* ≤ 0.0001).

**Table 1.** Clinical-demographic features of the study participants.

**Project**	**Smokers** (*N* = 177)	**Ex-smokers** (*N* = 63)	**No smokers** (*N* = 169)	***ANOVA***	
				***F***	***Sig*.**
**Male**	67	16	52	-	-
**Female**	110	47	117	-	-
**Age (years), *mean* (*SD*)**	30.45 (10.54)	35.33 (11.51)	32.50 (11.32)	4.56	**0.011**
**Educational level (years), *mean* (*SD*)**	15.31 (2.17)	15.86 (1.82)	15.67 (2.15)	2.09	0.124
Age of smoking onset, *mean* (*SD*)*	17.68 (4.38)	16.75 (2.96)	-	4.84	0.117
Attempts to stop smoking, *mean* (*SD*)*	1.89 (2.37)	2.51 (1.95)	-	2.40	0.272

Note: *student *T* test between smokers and ex-smokers.

**Table 2.** TEMPS-A and BPS mean scores in study participants.

**Project**	**Smoker** (*N* = 177)	**Ex-smoker** (*N* = 63)	**No smoker** (*N* = 169)	***ANOVA***	
	*Mean* (SD)	*Mean* (SD)	*Mean* (SD)	***F***	***Sig*.**
**TEMPS-A**					
Cyclothymic	5.23 (3.40)	4.00 (3.37)	3.56 (2.75)	12.563	**0.000**
Depressive	2.76 (2.20)	2.43 (2.08)	2.38 (2.15)	1.485	0.228
Irritable	1.49 (1.69)	1.17 (1.24)	1.11 (1.49)	2.793	0.062
Hyperthymic	4.24 (1.91)	3.81 (2.21)	3.77 (2.01)	2.700	0.068
Anxious	1.43 (1.03)	1.46 (1.15)	1.24 (1.09)	1.756	0.174
**BPS**					
Internal Stimulation Creativity	25.87 (6.30)	25.62 (6.36)	25.54 (6.10)	0.124	0.883
Apathy	18.06 (6.45)	17.33 (6.08)	17.78 (7.29)	0.279	0.757
External Stimulation Challenge	16.61 (5.53)	16.70 (5.26)	16.04 (5.51)	0.585	0.558

**Table 3.** Linear regression analysis.

Project		Unstandardized coefficients		Standardized coefficients	*t*	*p*
Dependent variable	Predictors	*B*	*S.E*.	*Beta*		
“Internal Stimulation Creativity”^a^ (Model 1)	(Constant)	21.430	1.220		17.57	0.000
	Age	0.440	0.250	0.790	1.740	0.082
	Cyclothymic	0.085	0.104	0.044	0.815	0.415
	Depressive	−0.665	0.165	−0.232	−4.050	**0.000**
	Irritable	−0.131	0.197	−0.033	−0.663	0.508
	Hyperthymic	1.160	0.142	0.376	8.170	**0.000**
	Anxious	−0.217	0.275	−0.038	−0.788	0.431
“Apathy”^b^ (Model 2)	(Constant)	15.170	1.260		12.030	0.000
	Age	−0.058	0.026	−0.095	−2.200	0.028
	Cyclothymic	0.567	0.108	0.271	5.270	**0.000**
	Depressive	0.909	0.170	0.292	5.330	**0.000**
	Irritable	0.142	0.204	0.033	0.696	0.487
	Hyperthymic	−0.178	0.147	−0.053	−1.210	0.227
	Anxious	0.196	0.285	0.031	0.687	0.492
“External Stimulation-Challenge”^c^ (Model 3)	(Constant)	12.070	1.100		10.970	0.000
	Age	−0.016	0.023	−0.032	−0.697	0.487
	Cyclothymic	0.618	0.094	0.365	6.580	**0.000**
	Depressive	0.235	0.149	0.093	1.580	0.114
	Irritable	0.263	0.178	0.075	1.480	0.140
	Hyperthymic	0.480	0.129	0.176	3.740	**0.000**
	Anxious	−0.525	0.249	−0.103	−2.110	0.035

Note: ^a^ *R* = 0.499; *F* = 22.174; *p* = 0.000; ^b^ *R* = 0.565; *F* = 31.397; *p* = 0.000; ^c^ *R* = 0.459; *F* = 17.915; *p* = 0.000

## 4. Discussion

We aim to explore the role of affective temperament as a predictor of boredom proneness, in an individual smoking cessation, and provides interesting insights into the role of boredom in the process of smoking cessation.

The first result of our study shows how smokers have the tendency for a cyclothymic temperament compared with non-smokers and compared with ex-smokers, which agrees with other researchers who found that cyclothymic, together with irritable people, showed a temperament predictor of self-reported nicotine abuse [28]. This data could explain as cyclothymic temperament contributes not only to cigarette smoking, but also to the difficulty to quit smoking. According to literature, ciclothymic temperament seems to have a correlation with novelty seeking, which was found to be related to smoking initiation in an adult sample. In a study of Bisol et al. [21], results showed how being more dysphoric, cyclothymic, labile, disinhibited, and irritable were particularly associated with a higher chance of being a current smoker. Although there is significant overlap between the general dimensions of personality and the concepts of boredom proneness, the latter is not completely explained by commonly measured personality traits.

Boredom proneness refers to an individual's inherent tendency to experience boredom. Previous studies have consistently demonstrated a strong association between this disposition and various negative outcomes. Nevertheless, our purpose has been to explore how the tendency towards boredom may interfere with the ability to quit smoking, in relation to affective temperaments. The tools of boredom proneness scale, used in this work, are explained as following: external stimulation is the individual's perceived need for environmental stimulation, such as excitement, novelty, and challenge; internal stimulation is an individual's ability to keep themselves interested and entertained; apathy is a lack of interest in one's environment [29]. The dimension of searching internal and external stimulation recall the concept of novelty seeking described as the tendency to desire novel stimuli and environments according to classical theories of temperaments [30],[31]. Human and rodent studies demonstrate that high novelty seeking can predict the initiation of drug use, including nicotine addiction. The reason is that both novelty seeking and addiction are modulated by the central reward system in the brain [30].

In our sample depressive temperament is inversely predictive of internal stimulation creativity, and directly predictive of apathy; this could mean that subjects with such temperament tend not to seek internal stimulation, rather manifesting a tendency toward an interest in environments. In the literature, other data claiming that cigarette smoking is over-represented in adult smokers prone to depression [32], while in our sample depressive temperament seems to be not so characterized among smokers. The factor that likely induces a subject with depressive temperament to smoke is not linked with the boredom dimensions.

Otherwise, hyperthymic temperament is directly predictive of internal stimulation creativity seeking, and external stimulation challenge seeking; thus, subjects with these temperaments experiment boredom if they are not able to generate interesting activities and if they experiment low environmental stimulation. According to literature, subjects with the hyperthymic temperament had a relatively low prevalence of smoking and were more likely to quit [21], considering this, and considering the dimension of boredom, if hyperthymic temperament manage to avoid experiencing a lack of internal and external stimuli, they can easily quit smoking.

On the same line, cyclothymic temperament is directly predictive of apathy and of external stimulation challenges, showing that these people tend to experiment boredom in situation of lack of interest in environment and, for this reason, they could use smoking as a solution to boredom. Once more for this kind of temperament, avoidance of boredom experiences can be valuable tool for therapeutic intervention on smoking cessation. Indeed, the pursuit of both internal and external sensations may be a contributing factor leading individuals to engage in persistent nicotine intake, thereby maintaining the activation of the reward circuit and creating a need for continuous stimulation [33]. In addition, the tendency to apathy in cyclothymic patients could be an additional obstacle to smoking cessation attempts and to maintaining abstinence. Moreover, during nicotine withdrawal, dopamine levels are significantly reduced, which is why people feel depressed after quitting smoking [34]. Being particularly susceptible to apathy could worsen this condition of abstinence depression, making resume smoking a coping strategy to alleviate the feelings of emptiness.

The present study should be interpreted in the light of the following limitations. First, the data was collected from a convenience sample, that is not representative of the general population, recruited through online procedure, and the cross-sectional design of the study do not allow generalization of data. Second, the use of short and anonymous self-report instruments could represent a bias. Finally, assessment of smoking habits was limited to two questions, and level of dependence was not explored.

## 5. Conclusion

Despite the limitations, our results support the view that boredom proneness could have a negative effect on difficulty to quit smoking in relationship of some kind of affective temperaments. In clinical practice, investigation of the boredom proneness should be considered for the choice of the treatment, and additional studies are warranted to continue researching whether boredom proneness is associated with the difficulty in quitting in smoking cessation interventions.

## Use of AI tools declaration

The authors declare they have not used Artificial Intelligence (AI) tools in the creation of this article.
